# A deep-learning system predicts glaucoma incidence and progression using retinal photographs

**DOI:** 10.1172/JCI157968

**Published:** 2022-06-01

**Authors:** Fei Li, Yuandong Su, Fengbin Lin, Zhihuan Li, Yunhe Song, Sheng Nie, Jie Xu, Linjiang Chen, Shiyan Chen, Hao Li, Kanmin Xue, Huixin Che, Zhengui Chen, Bin Yang, Huiying Zhang, Ming Ge, Weihui Zhong, Chunman Yang, Lina Chen, Fanyin Wang, Yunqin Jia, Wanlin Li, Yuqing Wu, Yingjie Li, Yuanxu Gao, Yong Zhou, Kang Zhang, Xiulan Zhang

**Affiliations:** 1 State Key Laboratory of Ophthalmology, Zhongshan Ophthalmic Center, Sun Yat-sen University, Guangdong Provincial Key Laboratory of Ophthalmology and Visual Science, Guangdong Provincial Clinical Research Center for Ocular Diseases, Guangzhou, China.; 2 State Key Laboratory of Biotherapy and Center for Translational Innovations, West China Hospital and Sichuan University, Chengdu, China.; 3 PKU-MUST Center for Future Technology, Faculty of Medicine, Macao University of Science and Technology, Macau, China.; 4 State Key Laboratory of Organ Failure Research, National Clinical Research Center for Kidney Disease and Nanfang Hospital, Southern Medical University, Guangzhou, China.; 5 Beijing Institute of Ophthalmology, Beijing Tongren Eye Center, Beijing Tongren Hospital, Beijing Ophthalmology and Visual Science Key Lab, Beijing, China.; 6 Department of Ophthalmology, Nanfang Hospital, Southern Medical University, Guangzhou, China.; 7 Department of Ophthalmology, Sichuan Academy of Medical Sciences & Sichuan Provincial People’s Hospital, Chengdu, China.; 8 Department of Ophthalmology, Guizhou Provincial People’s Hospital, Guiyang, China.; 9 Nuffield Laboratory of Ophthalmology, Department of Clinical Neurosciences, University of Oxford and Oxford University Hospitals NHS Foundation Trust, Oxford, United Kingdom.; 10 He Eye Specialist Hospital, Shenyang, Liaoning Province, China.; 11 Jiangmen Xinhui Aier New Hope Eye Hospital, Jiangmen, Guangdong, China.; 12 Department of Ophthalmology, Zigong Third People’s Hospital, Zigong, China.; 13 Department of Ophthalmology, Fujian Provincial Hospital, Fuzhou, China.; 14 Department of Ophthalmology and Optometry, Guizhou Nursing Vocational College, Guiyang, China.; 15 Department of Ophthalmology, Guangzhou Development District Hospital, Guangzhou, China.; 16 Department of Ophthalmology, The Second Affiliated Hospital of Guizhou Medical University, Kaili, China.; 17 Department of Ophthalmology, The Third People’s Hospital of Dalian, Dalian, Liaoning Province, China.; 18 Department of Ophthalmology, Shenzhen Qianhai Shekou Free Trade Zone Hospital, Shenzhen, China.; 19 Department of Ophthalmology, Dali Bai Autonomous Prefecture People’s Hospital, Dali, China.; 20 Department of Ophthalmology, Wuwei People’s Hospital, Wuwei, Gansu Province, China.; 21 Department of Ophthalmology, Joint Shantou International Eye Center of Shantou University and the Chinese University of Hong Kong, Shantou, Guangdong, China.; 22 Department of Ophthalmology, The First Hospital of Nanchang City, Nanchang, China.; 23 State Key Laboratory of Lunar and Planetary Sciences, Macao University of Science and Technology, Taipa, Macau, China.; 24 Clinical Research Institute, Shanghai General Hospital, Shanghai Jiaotong University School of Medicine, Shanghai, China.

**Keywords:** Ophthalmology, Translation

## Abstract

**Background:**

Deep learning has been widely used for glaucoma diagnosis. However, there is no clinically validated algorithm for glaucoma incidence and progression prediction. This study aims to develop a clinically feasible deep-learning system for predicting and stratifying the risk of glaucoma onset and progression based on color fundus photographs (CFPs), with clinical validation of performance in external population cohorts.

**Methods:**

We established data sets of CFPs and visual fields collected from longitudinal cohorts. The mean follow-up duration was 3 to 5 years across the data sets. Artificial intelligence (AI) models were developed to predict future glaucoma incidence and progression based on the CFPs of 17,497 eyes in 9346 patients. The area under the receiver operating characteristic (AUROC) curve, sensitivity, and specificity of the AI models were calculated with reference to the labels provided by experienced ophthalmologists. Incidence and progression of glaucoma were determined based on longitudinal CFP images or visual fields, respectively.

**Results:**

The AI model to predict glaucoma incidence achieved an AUROC of 0.90 (0.81–0.99) in the validation set and demonstrated good generalizability, with AUROCs of 0.89 (0.83–0.95) and 0.88 (0.79–0.97) in external test sets 1 and 2, respectively. The AI model to predict glaucoma progression achieved an AUROC of 0.91 (0.88–0.94) in the validation set, and also demonstrated outstanding predictive performance with AUROCs of 0.87 (0.81–0.92) and 0.88 (0.83–0.94) in external test sets 1 and 2, respectively.

**Conclusion:**

Our study demonstrates the feasibility of deep-learning algorithms in the early detection and prediction of glaucoma progression.

**FUNDING:**

National Natural Science Foundation of China (NSFC); the High-level Hospital Construction Project, Zhongshan Ophthalmic Center, Sun Yat-sen University; the Science and Technology Program of Guangzhou, China (2021), the Science and Technology Development Fund (FDCT) of Macau, and FDCT-NSFC.

## Graphical abstract

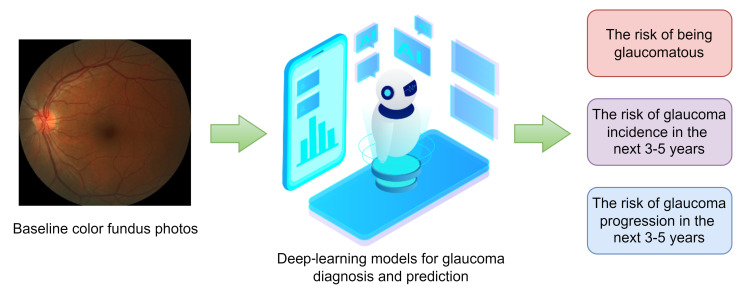

## Introduction

Glaucoma is a major chronic eye disease characterized by optic nerve damage and visual field defects (1, 2). Its onset is often insidious, with a risk of irreversible visual field loss prior to becoming symptomatic (3). Timely detection and treatment of glaucoma by lowering the intraocular pressure (IOP) could reduce the risk of disease progression (4, 5). Predicting glaucoma onset and progression is a major clinical challenge. Previous studies demonstrated that biometric parameters, such as baseline IOP, vertical cup-to-disc ratio, mean deviation (in the Humphrey visual field test), and pattern standard deviation are helpful in predicting glaucoma incidence and progression (6–12). However, IOP measurement and visual field tests are often not available in the primary healthcare setting. In contrast, color fundus photography is widely available and color fundus photographs (CFPs) can be rapidly acquired, with the potential to allow artificial intelligence–based (AI-based) diagnosis of optic nerve, retinal, and systemic diseases (including chronic kidney disease, diabetes mellitus; ref. 13). Smartphones can also be adapted to capture CFPs, making them a promising tool in disease screening in the future (14, 15). Thus, it would be advantageous if glaucoma incidence and progression could be solely based on CFPs rather than relying on multiple test modalities.

**Figure 1:**
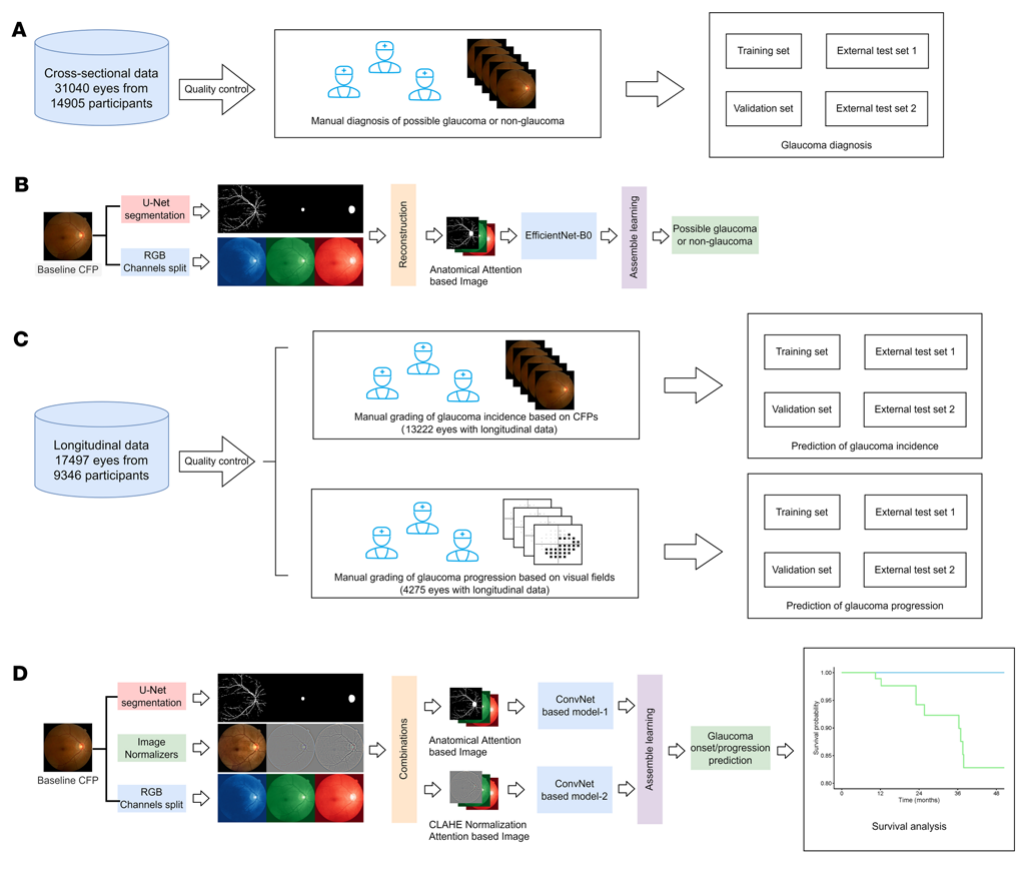
Development and validation of the deep-learning system for glaucoma diagnosis and incidence and progression prediction. (**A**) Data collection and ground truth labeling of glaucoma diagnosis based on CFPs. (**B**) Pipeline for glaucoma diagnosis. (**C**) Data collection and ground truth labeling of glaucoma incidence and progression. (**D**) Pipeline for predicting glaucoma development and progression. CFP, color fundus photograph; VF, visual field.

Deep-learning techniques have been widely used for glaucoma diagnosis (16–19). However, there is no clinically validated algorithm for glaucoma incidence and progression prediction. This study aimed to develop a clinically feasible deep-learning system for diagnosing glaucoma (Figure 1, A and B) and predicting the risk of glaucoma onset and progression (Figure 1, C and D) based on CFPs, with validation of performance in external population cohorts. Our AI system appears to be capable of detecting features in the baseline CFPs that are unrecognizable to the human eye and predict which patients will progress to glaucoma within 5 years. Furthermore, we show that the AI system could be deployed at the point of care via smartphone image capture to enable broadly accessible remote glaucoma screening in the future.

## Results

### Definitions of glaucoma, its incidence, and progression.

The diagnostic criteria for possible glaucoma based on CFPs were created following published population-based studies; glaucomatous optic neuropathy was defined by the presence of a vertical cup-to-disc ratio of 0.7 or greater, retinal nerve fiber layer (RNFL) defect, optic disc rim width of 0.1-disc diameter or smaller, and/or disc hemorrhage (20–22). Glaucoma incidence was defined as eyes having nonglaucomatous baseline CFPs but becoming possibly glaucomatous during a follow-up period.

Humphrey visual fields performed in a standard 24-2 pattern mode were used for an analysis when glaucoma progression was suspected (23). Glaucomatous progression was defined by at least 3 visual field test points worse than the baseline at the 5% level in 2 consecutive reliable visual field tests or at least 3 visual field locations worse than the baseline at the 5% level in 2 subsequent consecutive reliable visual field tests (23). Time to progression was defined as the time from a baseline to the first visual field test report that confirmed glaucoma progression following the aforementioned criteria. The gold standard definition of clinical progression was confirmed to have been met by unanimous agreement of 3 ophthalmologists who independently assessed each visual field report.

### Image data sets and patient characteristics.

We established a large data set composed of CFPs and visual fields collected in Guangzhou, Beijing, and Kashi, China. The demographic and clinical information of the study participants is summarized in Table 1. The data were split randomly into mutually exclusive sets for training, validation, and external testing of the AI algorithms.

In the first task, we developed a model to diagnose possible glaucoma based on 31,040 CFPs. In this task, 31,040 images (split into 20,872 for training, 3182 for validation, 6162 for external test 1,and 824 for external test 2) from 14,905 individuals were collected from glaucoma and anterior segment disease eye clinics. Among these images, 10,175 (32.8%) were diagnosed with possible glaucoma. The training and validation data sets were obtained from individuals from glaucoma and anterior segment disease sections in the Zhongshan Ophthalmic Center in Guangzhou, China. External test set 1 was collected from patients in the glaucoma and anterior segment disease clinic in Jidong Hospital near Beijing. To further test the generalizability of the AI model, we validated its performance with CFPs obtained by smartphones from Kashi.

In the second task, we developed a model to predict future glaucoma incidence based on the data from 3 longitudinal cohorts. We included a total of 13,222 eyes (10,357 training, 1191 validation, 955 external test 1, 719 external test 2) of 7127 participants, all of which were diagnosed as nonglaucomatous at the baseline. The training and validation data sets were obtained from individuals who underwent an annual health check in Guangzhou, while external test set 1 was from individuals who underwent an annual health check in Beijing and external test set 2 was from a community cohort in Guangzhou. The mean follow-up duration was 47.8–56.6 months across the data sets. The incidence rate of glaucoma was 1.1%–2.0% across the data sets.

In the third task, we developed a model to predict glaucoma progression based on the CFPs from cohorts with existing glaucoma. In this task, 4275 eyes (3003 training, 422 validation, 337 external test 1, 513 external test 2) from 2219 glaucoma patients were included, all of which were already diagnosed with glaucomatous optic neuropathy at the baseline. The training and validation data sets were obtained from 1 primary open-angle glaucoma (POAG) cohort in the Zhongshan Ophthalmic Center. To further test the generalizability of the AI model on different subtypes of glaucoma, external test set 1 was collected from another POAG cohort and external test set 2 was collected from a chronic primary angle-closure glaucoma (PACG) cohort in the Zhongshan Ophthalmic Center. The mean follow-up duration was 34.8–41.7 months across the data sets, and the proportion of glaucoma progression was 6%–13.5% across the data sets (Table 1).

### Design of the diagnostic (DiagnoseNet) and predictive (PredictNet) algorithms.

First, we developed a diagnostic algorithm for possible glaucoma, DiagnoseNet (Figure 1B). In brief, DiagnosetNet is composed of 2 main modules, a segmentation module and a diagnostic module. The CFPs were semantically segmented by the segmentation module with 4 anatomical structures: retinal vessels, macula, optic cup, and optic disk. The diagnostic module generated the glaucomatous probability score.

We then designed a pipeline, PredictNet, for incidence and progression prediction of glaucoma. In brief, PredictNet is also composed of 2 main modules, the segmentation module and the prediction module. The segmentation module is the same as that in DiagnoseNet. The prediction module produces the risk score of glaucoma incidence or progression in the future (Figure 1D and Supplemental Figure 1).

The diagnostic and predictive algorithms share the same segmentation module. The segmentation module was trained based on manual annotations of optic disc (1853 images), optic cup (1860 images), macula (1695 images), and blood vessels (160 images) independently. The segmentation module demonstrated outstanding segmentation performance on the above anatomical structures and achieved an intersection over union (IOU) of 0.847, 0.669, 0.570, and 0.538 for optic disc, optic cup, macula, and blood vessel segmentation, respectively (Supplemental Table 1). Representative samples of segmentation are shown in Supplemental Figure 2.

### Diagnostic performance of the AI model based on CFPs captured by smartphones.

To demonstrate the potential of deploying our AI model in routine healthcare, we developed and tested the AI model to diagnose possible glaucoma based on CFPs not only from fundus cameras but also from smartphones. As shown in Table 2, in this validation data set, the AI model achieved an area under the receiver operating characteristic (AUROC) curve of 0.97 (0.96–0.97), a sensitivity of 0.98 (0.97–0.99), and a specificity of 0.82 (0.80–0.83) for differentiating glaucomatous and nonglaucomatous eyes. To evaluate the generalizability of the algorithms, the AI model was tested on 2 external data sets. In external test set 1, the AI model achieved an AUROC of 0.94 (0.93–0.94), a sensitivity of 0.89 (0.87–0.90), and a specificity of 0.83 (0.81–0.84). In external test set 2, which was obtained using smartphones, the AI model achieved an AUROC of 0.91 (0.89–0.93), a sensitivity of 0.92 (0.88–0.96), and a specificity of 0.71 (0.67–0.74).

### Prediction of glaucoma incidence using longitudinal cohorts.

**Figure 2:**
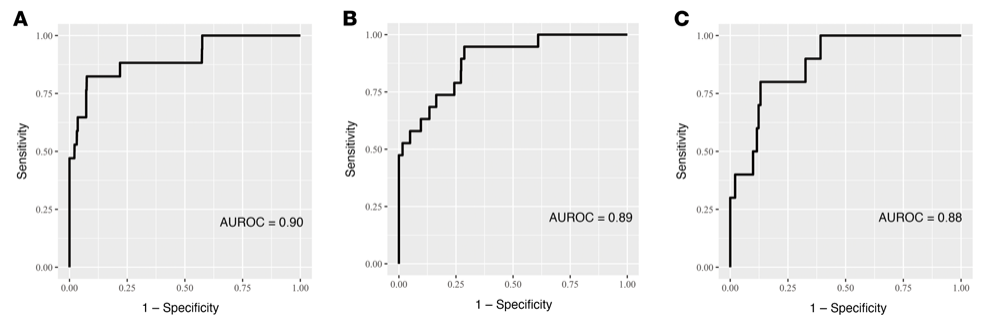
Area under the receiver operating characteristic (AUROC) curves of the AI model for prediction of glaucoma onset. (**A**–**C**) Predictive performance of the AI model in the validation set (*n =* 1191), external test set 1 (*n =* 955), and external test set 2 (*n =* 719).

We investigated the predictive performance of the AI model for the development of glaucoma in nonglaucomatous individuals over a 4- to 5-year period. A total of 158 eyes developed glaucoma within the 4- to 5-year period. The AI model achieved an AUROC of 0.90 (0.81–0.99), a sensitivity of 0.84 (0.82–0.87), and a specificity of 0.82 (0.57–0.96) for predicting glaucoma incidence in the validation set (Table 2 and Figure 2). The AI model demonstrated good generalizability in the external test sets, which achieved an AUROC of 0.89 (0.83–0.95), a sensitivity of 0.84 (0.81–0.86), and a specificity of 0.68 (0.43–0.87) in external test set 1, and an AUROC of 0.88 (0.79–0.97), a sensitivity of 0.84 (0.81–0.86), and a specificity of 0.80 (0.44–0.97) in external test set 2 (Table 2, Figure 2, and Supplemental Figure 3).

Supplemental Table 2 shows the incidence of glaucoma stratified by the AI model. As shown in Supplemental Table 2, there was a significant difference in the incidence rate of glaucoma between the low-risk and high-risk groups. The incidence rates were 0.2% and 5.0%, 0.6% and 5.6%, and 0.4% and 4.1% in the low- and high-risk groups of the validation set, external test set 1, and external test set 2, respectively. We employed the Kaplan-Meier approach to stratify healthy individuals into 2 risk categories (low or high risk) for developing glaucoma, based on 4- to 5-year longitudinal data on glaucoma development. The upper quartile of the predicted risk scores from the model in the validation set was used to create the threshold for the high-risk and low-risk groups in the Kaplan-Meier curves and log-rank tests. In the external test sets, significant separations of the low- and high-risk groups were achieved (both *P* < 0.001, Supplemental Figure 4).

The distribution of the risk scores and the threshold (upper quartile) of low- and high-risk groups across the validation and external test sets are presented in Supplemental Figure 5. As shown in the figure, the threshold (risk score of 0.3561, black dotted line) well defines a boundary to separate individuals who are likely and unlikely to develop glaucoma in a 4- to 5-year period.

Supplemental Table 3 presents the results of subgroup analyses within the validation and external test sets. The AI model demonstrated no statistically significant difference in performance among the subgroups as stratified by age (≥60 vs. <60 years), sex (male vs. female), and severity of glaucoma (mean deviation > –6 dB vs. < –6 dB).

### Prediction of the glaucoma progression using longitudinal cohorts.

**Figure 3:**
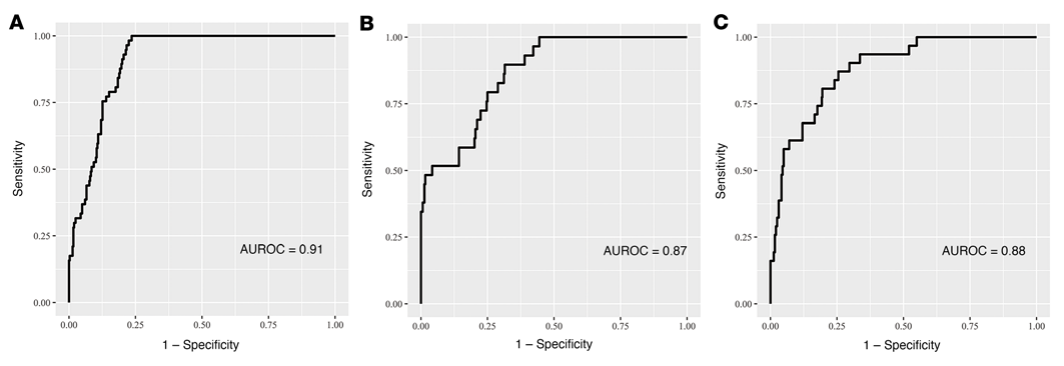
Area under the receiver operating characteristic (AUROC) curves of the AI model for prediction of glaucoma progression. (**A**–**C**) Predictive performance of the AI model in the validation set (*n =* 422), external test set 1 (*n =* 337), and external test set 2 (*n =* 513).

We investigated the predictive performance of the AI model for glaucoma progression in glaucomatous eyes over a 3- to 4-year period. A total of 444 POAG eyes had progression within the 3- to 4-year period. The AI model achieved an AUROC of 0.91 (0.88–0.94), a sensitivity of 0.83 (0.79–0.87), and a specificity of 0.79 (0.66–0.89) for predicting glaucoma progression in the validation set (Table 2 and Figure 3). To validate the generalizability of the AI model in predicting progression in multiple-mechanism glaucoma, we further tested its predictive performance in 2 independent cohorts of PACG (external test set 1) and POAG (external test set 2). The AI model achieved excellent predictive performance, with an AUROC of 0.87 (0.81–0.92), a sensitivity of 0.82 (0.78–0.87), and a specificity of 0.59 (0.39–0.76) in external test set 1, and an AUROC of 0.88 (0.83–0.94), a sensitivity of 0.81 (0.77–0.84), and a specificity of 0.74 (0.55–0.88) in external test set 2 (Table 2, Figure 3, and Supplemental Figure 6).

We also trained a predictive model using baseline clinical metadata (age, sex, intraocular pressure, mean deviation, pattern standard deviation, and hypertension or diabetes status) alone to predict progression, which led to an AUROC of 0.76 (0.70–0.82), 0.73 (0.66–0.79), and 0.44 (0.33–0.54) in the validation set, external test set 1, and external test set 2, respectively (Supplemental Figure 7). The performance of the AI model was significantly better than that of the predictive model based on baseline metadata in the above data sets (all *P* < 0.001).

Supplemental Table 4 shows the risk of glaucoma progression stratified by the AI model. As shown in Supplemental Table 4, there was a significant difference in the proportion of eyes with glaucoma progression in the low-risk and high-risk groups. The incidence rates were 3.8% and 42.4%, 4.5% and 23.9%, and 2.0% and 19.8% in the low and high-risk groups of the validation set, external test set 1, and external test set 2, respectively. We then performed Kaplan-Meier analysis to stratify glaucomatous eyes into 2 risk categories (low or high risk) for glaucoma progression, based on 3- to 4-year longitudinal data on glaucoma progression. The upper quartile of the predicted risk scores from the model in the validation set was used to create the threshold for the high-risk and low-risk groups in the Kaplan-Meier curves and log-rank tests. In the external test sets, significant separations of the low- and high-risk groups were achieved (both *P* < 0.001, Supplemental Figure 8).

The distribution of the risk scores and the threshold (upper quartile) of low- and high-risk groups across the validation and external test sets are presented in Supplemental Figure 9. As shown in the figure, the threshold (risk score of 2.6352, black dotted line) well defines a boundary to separate glaucomatous eyes that are likely and unlikely to progress in a 3- to 4-year period.

Supplemental Table 3 presented the results of the subgroup analysis in the validation and external test sets. The AI model demonstrated no statistical significance in all the subgroups stratified by age (≥60 vs. <60 years), sex (male vs. female), and severity of glaucoma (mean deviation > –6 dB vs. < –6 dB) except the AUROCs of severe and less severe subgroups in the validation set and external test set 1.

### Visualization of the evidence for prediction of glaucoma incidence and progression.

**Figure 4:**
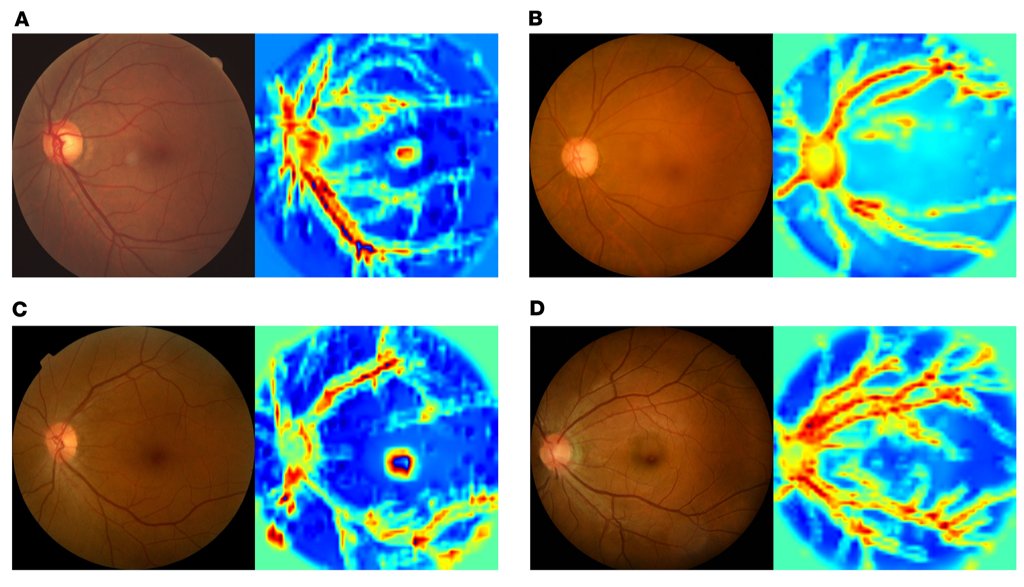
Saliency maps of the deep-learning models. Visual explanation of the key regions the models used for diagnostic predictions. (**A** and **B**) The heatmaps of the typical samples of eyes with (**A**) and without (**B**) glaucoma development. (**C** and **D**) The heatmaps of the typical samples of eyes with (**C**) and without (**D**) glaucoma progression. In both tasks, the saliency maps suggest that the AI model focused on the optic disc rim and areas along the superior and inferior vascular arcades, which are consistent with the clinical approach whereby nerve fiber loss at the superior or inferior disc rim provides key diagnostic clues. AI-based predictions also appear to involve the retinal arterioles and venules.

To improve the interpretability of the AI models and illustrate the key regions for AI-based predictions, we used gradient-weighted class activation mapping (Grad-CAM) to generate the key regions in the CFPs for diagnosing glaucoma and predicting glaucoma incidence and progression. Representative cases and their corresponding saliency maps of DiagnoseNet are presented in Supplemental Figure 10. Representative cases and their corresponding saliency maps are presented in Supplemental Figure 10 (DiagnoseNet) and Figure 4 (PredictNet). The saliency maps suggest that the AI model focused on the optic disc rim and areas along the superior and inferior vascular arcades, which is consistent with the clinical approach whereby nerve fiber loss at the superior or inferior disc rim provides key diagnostic or predictive clues. This would suggest that the AI models are learning clinically relevant knowledge in evaluating glaucoma diagnosis and progression. AI-based predictions also appear to involve the retinal arterioles and venules, thus implicating vascular health as potentially relevant to the etiology of chronic open-angle glaucoma.

## Discussion

More than 60 million people in the world suffer from glaucoma, and the number is predicted to increase to 110 million by 2040 (24). Due to its insidious onset and variable progression, diagnosis of glaucoma and monitoring of treatment can be challenging and clinically time consuming. Glaucoma screening is not universal around the world, thus leading to a delayed diagnosis and severe irreversible sight loss. Therefore, there is a high clinical demand for an efficient and reliable AI model to help identify high-risk individuals for glaucoma development and progression within the population in order to facilitate early intervention.

Deep-learning algorithms have been widely used in glaucoma diagnostic studies (16–19), and have achieved outstanding diagnostic performance in detecting glaucomatous eyes. However, few studies have explored the efficacy of deep learning in glaucoma onset and progression prediction (25–29). In this study, our AI model showed excellent glaucoma diagnostic performance on CFPs, including photographs captured with smartphone cameras using an adaptor, which could significantly broaden its application at a point-of-care setting. Compared with traditional statistical models (30–33), such as glaucoma probability score and Moorfields regression analysis, several studies using deep-learning models achieved comparable or even better predictive performance (25–27). Thakur et al. developed AI models to predict glaucoma development approximately 1 to 3 years before clinical onset and achieved a highest AUROC of 0.88 (25). However, these deep-learning models had some limitations. First, the application was limited to onset prediction without progression prediction, the latter being an essential part of glaucoma management. Second, the data mostly came from hospitals or clinical trials rather than community populations, including many eyes that were diagnosed with ocular hypertension (elevated intraocular pressure without optic neuropathy) rather than glaucoma (25). Third, there is a lack of external validation data to demonstrate the generalizability of the model in the community.

Compared with previous studies, our study has the following advantages. First, we developed AI models for glaucoma diagnosis and incidence and progression prediction. In the external test sets, the models achieved excellent predictive performance in identifying high-risk individuals for developing glaucoma or having glaucoma progression. Secondly, data in glaucoma incidence prediction came from community screening settings, which better reflects the distribution characteristics of glaucoma in the population and facilitates the generalizability of the model. The results in the external data sets show the AI model achieved an excellent predictive performance of glaucoma development, demonstrating strong generalizability and reliability of the AI model. Third, all the patients in the glaucoma cohorts of the progression prediction task have received IOP-lowering medications since enrollment and their IOP values were all controlled within a normal range. This indicates that our predictive model could identify high-risk patients who will undergo glaucoma progression even with reasonably controlled IOPs and facilitate timely interventions such as antiglaucoma surgeries to save vision. Fourth, the AI model based on structural data from CFPs achieved a high predictive accuracy of glaucoma progression, as determined by the gold standard of visual field test results. Visual field tests can reveal functional damage of the optic nerve and are the clinical gold standard in monitoring glaucoma progression (34). As demonstrated in the task of glaucoma progression prediction, the AI model succeeded in identifying the high-risk eyes of progressive functional deterioration from baseline CFPs with high sensitivities. In addition, the AI model showed a similar predictive performance in different subtypes of glaucoma, including POAG and PACG, which share similar structural and functional damage of the optic nerve.

Our study has the following limitations. First, the input data of our AI models are only CFPs. Clinical glaucoma evaluation generally requires integrated analysis of multiple modalities (e.g., clinical examination, optic nerve head imaging, and visual field testing) to determine the glaucoma subtypes and any progression. Our study chose CPFs as the only input due to their high feasibility and widespread availability. Future studies may consider incorporating other data modalities to further improve the predictive performance of the algorithms. Second, only high-quality CFPs were included in the study, which limits the application of the AI models in eyes with media opacities that prevent obtaining clear CFPs. Third, limited by the prevalence of glaucoma in the general population (around 1% to 1.5% in those 40 to 65 years old) (35), there was a relatively small number of cases of glaucoma. To address this issue, we used a deep-learning model with relatively few parameters. Fourth, the AI models presented varied sensitivity and specificity across the data sets, although they had high AUROC values. High sensitivity is more important for screening, and we may further improve the predictive performance of the AI models with more training data in the future. Fifth, all the data were from the Chinese population and further validation is needed in other populations.

In conclusion, our study demonstrates the feasibility of deep-learning systems for disease onset and progression prediction. It offers the possibility of building a virtual glaucoma screening system in the future.

## Methods

### Data set characteristics

#### Glaucoma diagnosis cohorts.

In these initial cohorts, we were specifically looking for patients visiting ophthalmologists who subspecialize in both glaucoma and anterior segment diseases. The population of patients seen by these ophthalmologists was highly enriched with POAG patients (36, 37). We purposely chose these initial cohorts to ensure that we were able to collect sufficient POAG patients as well as nonglaucomatous control patients (such as cataract patients) who were otherwise appropriately matched for developing an AI-based diagnosis of POAG (Table 1). The training and validation data in glaucoma diagnosis were collected from community cohorts and eye clinics in Guangzhou. To test the generalizability of the AI model, 2 independent data sets obtained from Beijing and Kashi were used as external test sets. The external test set 1 was collected from patients who underwent an annual health check in Beijing city, while the external test set 2 was obtained by smartphones from local eye clinics in Kashi in the Xinjiang Autonomous Region.

#### Glaucoma incidence prediction cohorts.

The training and validation data in the prediction of glaucoma incidence were collected from community cohorts in Guangzhou. To test the generalizability of the AI model, 2 independent data sets obtained from Beijing and Guangzhou communities were used as external test sets. Our longitudinal cohorts for POAG incident prediction had POAG frequencies of around 1% to 2%, which is well within the norm of the prevalence of POAG in the general population.

#### Glaucoma progression prediction cohorts.

The training and validation data in predicting glaucoma progression were collected from 1 POAG cohort in the Zhongshan Ophthalmic Center, Guangzhou. To test the generalizability of the AI model, 2 independent cohorts composed of PACG and POAG eyes from the Zhongshan Ophthalmic Center were used as external test sets.

### Image quality control and labeling

Supplemental Figure 11 describes the data sets used in this study and the process of image quality control. All of the images were first deidentified to remove any patient-related information. Fifteen ophthalmologists with at least 10 years of clinical experience were recruited to label the CFPs. First, they were asked to exclude the images with poor quality. The criteria include (a) optic disc or macula was not fully visible and (b) blurred images due to refractive media. A fraction of the CFPs (7.1%) was excluded due to poor quality. Second, the graders were asked to assign glaucoma or nonglaucoma labels to each CFP. Third, each glaucomatous eye with longitudinal follow-up data was further analyzed to determine whether there was a progression based on the visual field reports during follow-up visits. Visual fields with fixation loss lower than 20%, a false positive rate lower than 15%, and a false negative rate lower than 33% were included. Each CFP or visual field report was evaluated by 3 ophthalmologists independently and the ground truths were determined by the consensus of 3 ophthalmologists.

### Criteria of glaucoma diagnosis and progression

Glaucoma was diagnosed using the criteria in previous population-based studies (20–22). Glaucomatous optic neuropathy was defined as the presence of vertical cup-to-disc ratio of 0.7 or greater, RNFL defect, optic disc rim width of 0.1-disc diameter or smaller, and/or disc hemorrhage. An eye would be labeled as possible glaucoma if one of the above criteria was met.

Glaucoma progression was determined based on the changes in the visual fields (23). The Humphrey Field Analyzer was used to perform all the visual field tests in 24-2 standard mode (Carl Zeiss Meditec). At least 3 visual field locations worse than baseline at the 5% level in 2 consecutive reliable visual fields, or at least 3 visual field locations worse than baseline at the 5% level in 2 consecutive reliable visual fields, were considered as progression (23). The time of progression was defined as the time from baseline to the first visual field that confirmed progression. Three ophthalmologists examined each visual field report separately to determine progression.

### Manual segmentation of anatomical structures

We randomly selected 2000 CFPs for manual segmentations of anatomical structures, including optic disc, optic cup, macula, and blood vessels. Two ophthalmologists independently annotated the CFPs at pixel level, and the final standard reference of annotations was determined by the mean of these 2 independent annotations.

### Model design of glaucoma prediction and ocular disease diagnosis

First, we developed an AI model, DiagnoseNet, to identify CFPs as glaucoma or nonglaucoma. DiagnoseNet is a pipeline composed of modules for segmentation and diagnosis. The fundus images were first semantically segmented in the segmentation module using U-Net (38) to produce 4 anatomical structures: retinal vessels, macula, optic cup, and optic disk. The segmentation data were then merged into a 1-channel by element-wise bit or operation over the 4 anatomical structure–focusing attention layers, which took the place of the CFPs’ blue channel to form a new CFP image. The diagnostic module’s backbone is EfficientNet-B0, with the last fully connected layer replaced by a Dense layer of 2 output units initialized with a random value, and the other layers’ initial weights determined from ImageNet’s pretrained settings (Figure 1B).

Then, we created a pipeline, PredictNet, to predict glaucoma onset and progression. PredictNet preprocesses and analyzes the CFP data (Supplemental Figure 1). First, in the preprocessing stage, the original fundus images are enhanced with contrast-limited adaptive histogram equalization (CLAHE) and color normalization (NORM). Important retinal structures, including optic disc, optic cup, macula, and blood vessels are semantically segmented with trained U-Net (38). The multiple-channel anatomical masks generated by U-Net are merged into a 1-channel mask and then fused with the green and red channels of CLAHE images to form CLAHE Normalization Attention–based images. NORM images are fused with the green and red channels of the original images to form anatomical attention–based images. Second, in analyzing stage, CLAHE Normalization Attention–based images and anatomical attention–based images are fed into 2 convolutional neural networks, namely ConvNet-based models 1 and 2. Each ConvNet-based model consists of a feature extraction network and a classification network module. The feature extraction network consists of 3 convolutional blocks, which are composed of a Convolution2D layer, a Batch Normalization layer, a LeakReLu layer, and a MaxPooling2D layer in series, while the classification network consists of 2 Dense layers in series. The GlobalMaxPooling2D layer is used to connect the feature extraction network and the classification network module. The final prediction is obtained by integrating the 2 ConvNet-based models in a linear combination. In the final step, PredictNet will generate a probability (*P*) of glaucoma incidence or progression between 0 and 1. *P* was transformed into a *z* score with the formula *z* score = (*P* – *P*′)/(standard deviation of *P*), where *P*′ is the mean *P* of each data set. Then, we obtained the final standard score by adding 1 to all the *z* scores, because some of the *z* scores were below zero.

The models were developed with Python (version 3.8.6; https://www.python.org/) and TensorFlow (version 2.1.0; https://github.com/tensorflow/tensorflow). The curves of training loss for each model were generated using TensorBoard (https://github.com/tensorflow/tensorboard) and are presented in Supplemental Figure 12. The key hyperparameters and average running time of each model are summarized in Supplemental Table 5.

### Interpretation of the AI model

Grad-CAM (39) was used to highlight the class-discriminative region in the images for predicting the decision of interest. We created heatmaps generated from CFPs, which indicated the key regions for the AI model to classify the CFPs into low- and high-risk groups.

### Data availability

Deidentified data may be available for research purposes from the corresponding authors on reasonable request.

### Statistics

The demographic characteristics of study participants are presented as mean ± SD for continuous data, and frequency (percentage) for categorical variables. AUROCs with 95% confidence interval (CI), sensitivity, and specificity were implemented to assess the performance of the algorithms. Sensitivity and specificity were determined by the selected thresholds in the validation sets. The survival curves were constructed for different risk groups, and the significance of differences between groups was tested by log-rank tests. The predictive performance of the AI model and metadata model was performed using DeLong’s test. All the hypotheses tested were 2-sided, and a *P* value of less than 0.05 was considered significant. All statistical analyses were performed using R (version 4.0; https://www.r-project.org/).

### Study approval

Institutional review board and ethics committee approvals were obtained in all locations and all the participants signed a consent form. All the images were uploaded to a Health Insurance Portability and Accountability Act–compliant cloud server for further grading.

### Code availability

The deep-learning models were developed and deployed using standard model libraries and the TensorFlow framework (version 2.3.0). Custom codes were specific to our development environment and used primarily for data input/output and parallelization across computers and graphics processors. The codes are available for research purposes from the corresponding authors on reasonable request.

## Author contributions

All authors collected and analyzed the data. KZ and XZ conceived and supervised the project. ZK, ZX, and LF wrote the manuscript. All authors discussed the results and reviewed the manuscript.

## Supplementary Material

Supplemental data

ICMJE disclosure forms

## Figures and Tables

**Table 1:**
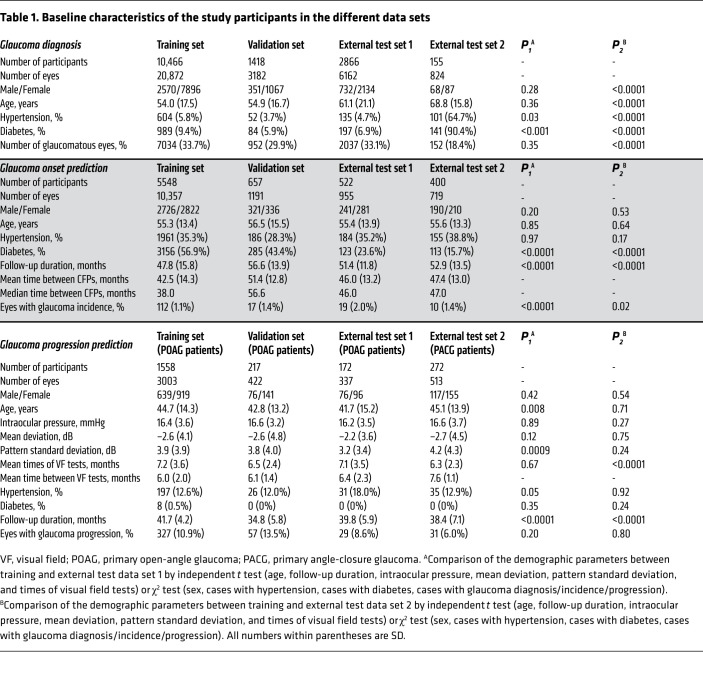
Baseline characteristics of the study participants in the different data sets

**Table 2:**
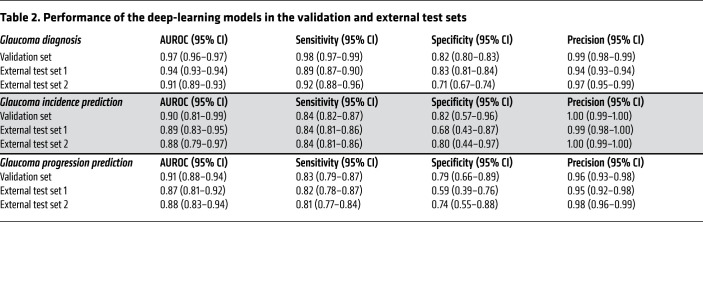
Performance of the deep-learning models in the validation and external test sets

## Version 1 06/01/2022

Electronic publication
