# Supplementary material for: A deep-learning system predicts glaucoma incidence and progression using retinal photographs
Source: J Clin Invest. 2022 Jun 1;132(11):e157968. doi: 10.1172/JCI157968 (PMC9151694; doi:10.1172/JCI157968)
Supplement: Supplemental data [file jci-132-157968-s094.pdf]

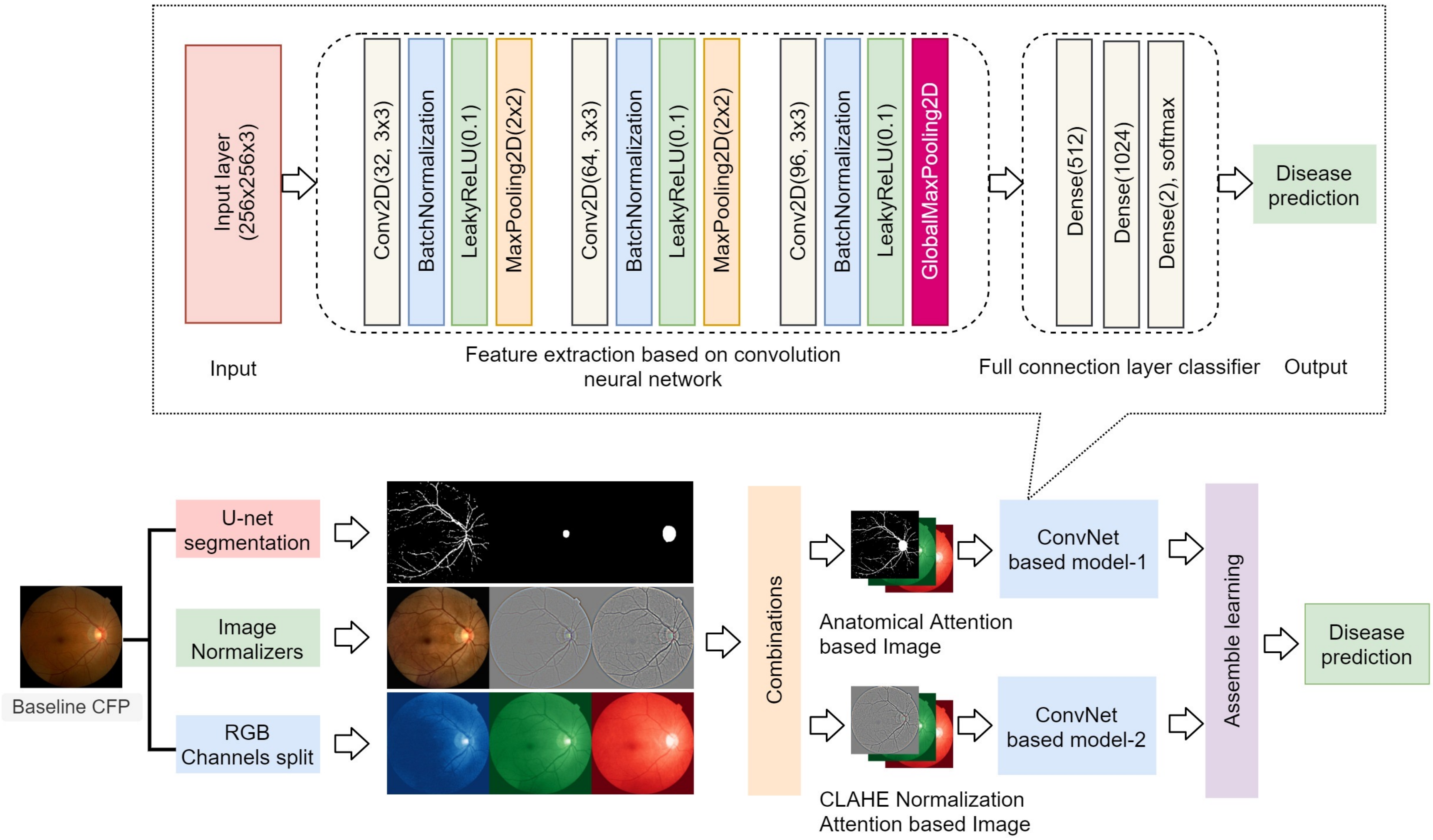

Supplementary Figure 1. Detailed architecture of PredictNet

PredictNet is composed of image preprocessing and analyzing modules. First, in preprocessing stage, the original fundus images are enhanced with Contrast Limited Adaptive Histogram Equalization (CLAHE) and color normalization (NORM). Important retinal structures, including optic disc, optic cup, macula and blood vessels are semantically segmented with trained Unet. The multi-channel anatomical masks output from the Unet are merged into a one-channel mask and then fused with the green and red channels of CLAHE images to form CLAHE Normalization Attention-based images. NORM images are fused with the green and red channels of the original images to form Anatomical Attention-based Images. Second, in analyzing stage, CLAHE Normalization Attention-based images and Anatomical Attention-based Images are fed into two convolutional neural networks, namely ConvNet based model 1 and 2. The final prediction is obtained by integrating the two ConvNet based models in a linear combination.

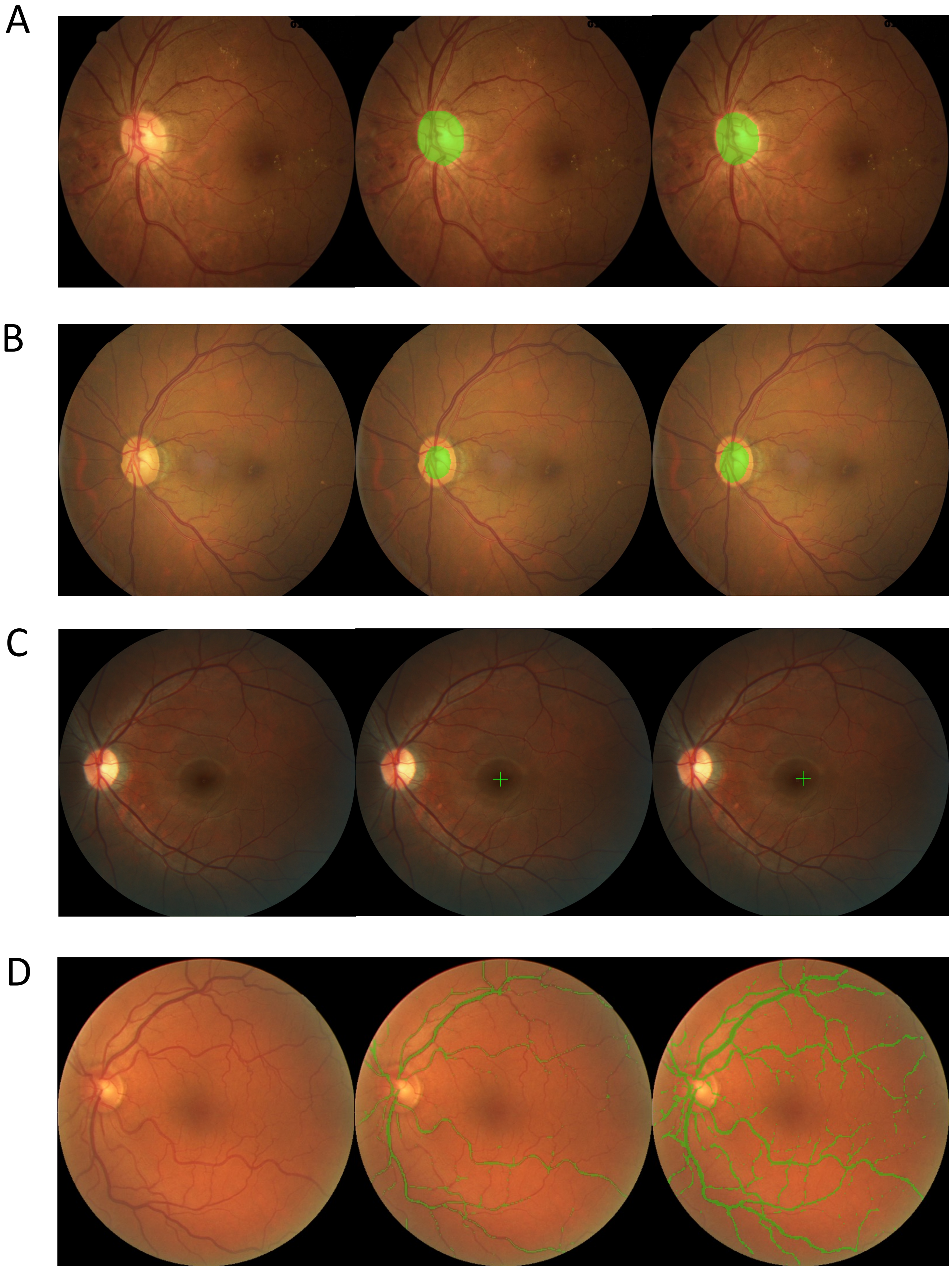

Supplementary Figure 2. Representative samples of automatic segmentation of optic disc, optic cup, macula and blood vessels

a to d: segmentation of optic disc, optic cup, macula and blood vessels. From left to right: original images, manual segmentations, automatic segmentations

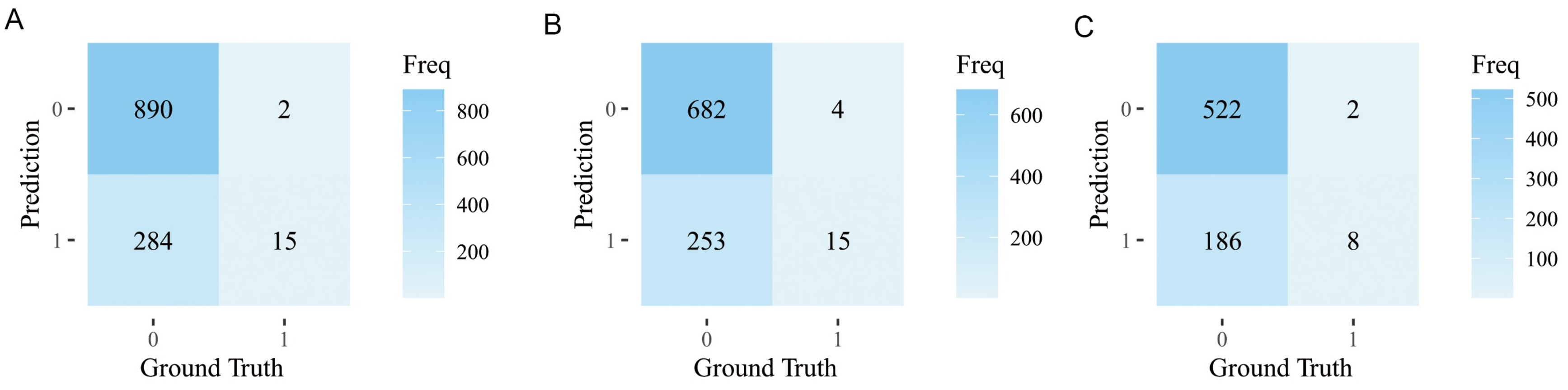

Supplementary Figure 3. Confusion matrices showing the predictive accuracy of the model across the datasets in the prediction of glaucoma onset

a to c: predictive accuracy in the validation set, and external test set 1, 2. 0 and 1 are labels for eyes without and with glaucoma incidence, respectively.

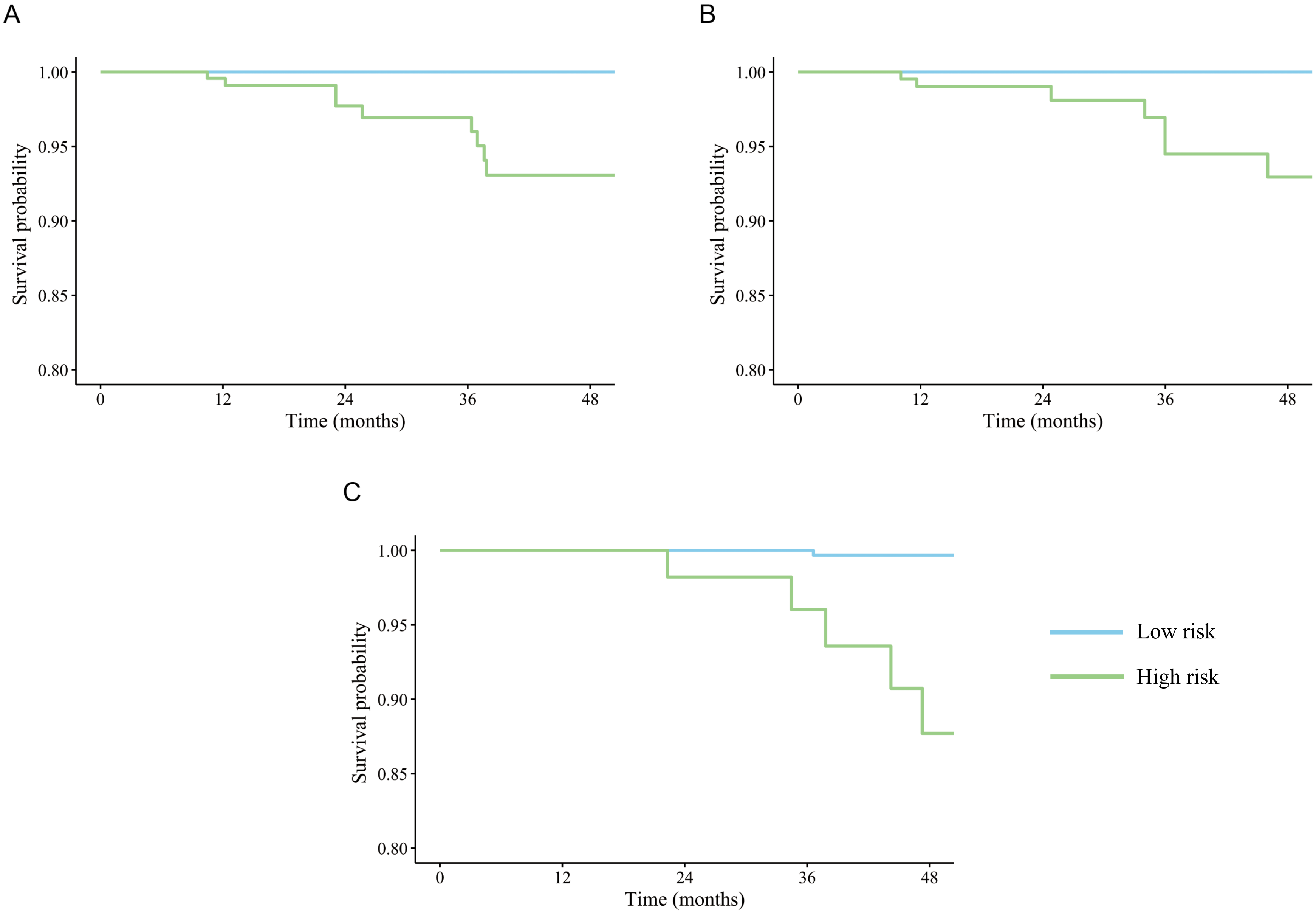

Supplementary Figure 4. Kaplan-Meier curves for predicting glaucoma development accuracy

a to c: predictive accuracy in the validation set, and external test set 1, 2. Survival curves in blue and green represent the high-risk and low-risk subgroups stratified by the upper quartile. P value is computed using a one-sided log-rank test between the two subgroups, and all P values are less than 0.001.

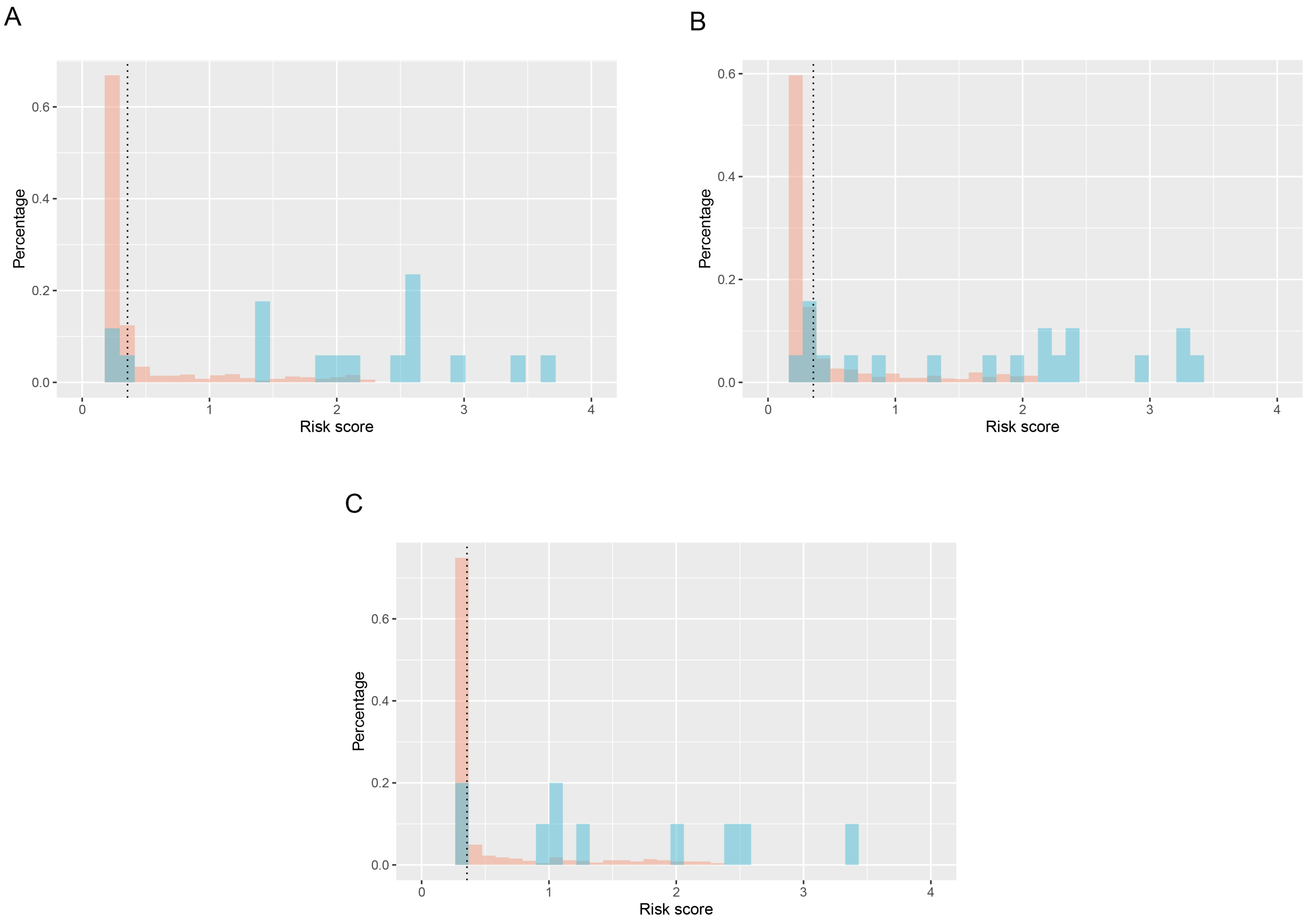

Supplementary Figure 5. The distribution of risk scores of the predictive models across all datasets in the prediction of glaucoma onset

The black dot line represents the low-high threshold of risk score (0.3561). The red bars represent the proportion of eyes without glaucoma development, while the blue bars represent the proportion of eyes with glaucoma development. a to c: glaucoma onset in the validation set, and external test set 1, 2.

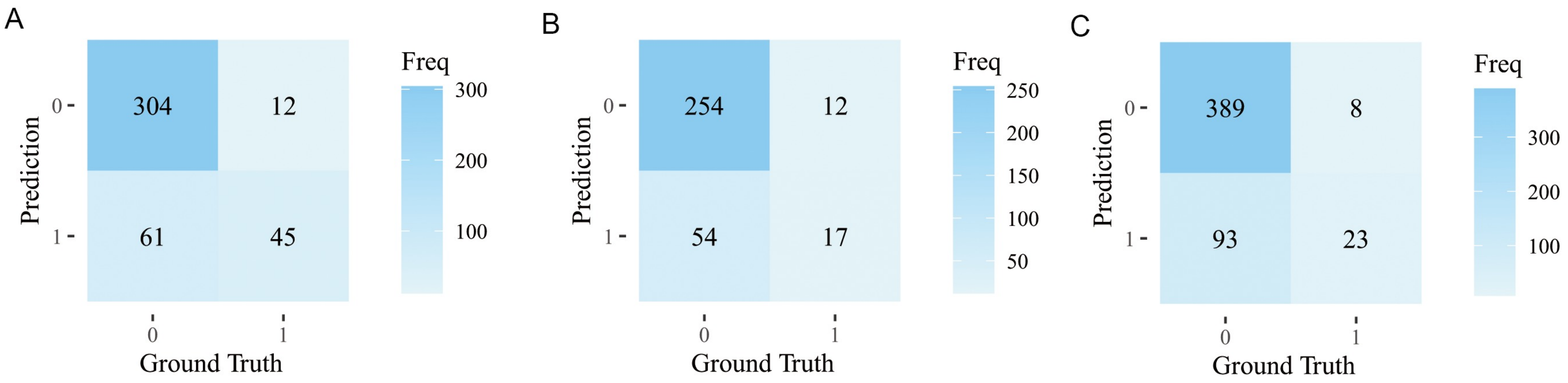

Supplementary Figure 6. Confusion matrices showing the predictive accuracy of the model across the datasets in the prediction of glaucoma progression

a to c: predictive accuracy in the validation set, and external test set 1, 2. 0 and 1 are labels for eyes without and wit glaucoma progression, respectively.

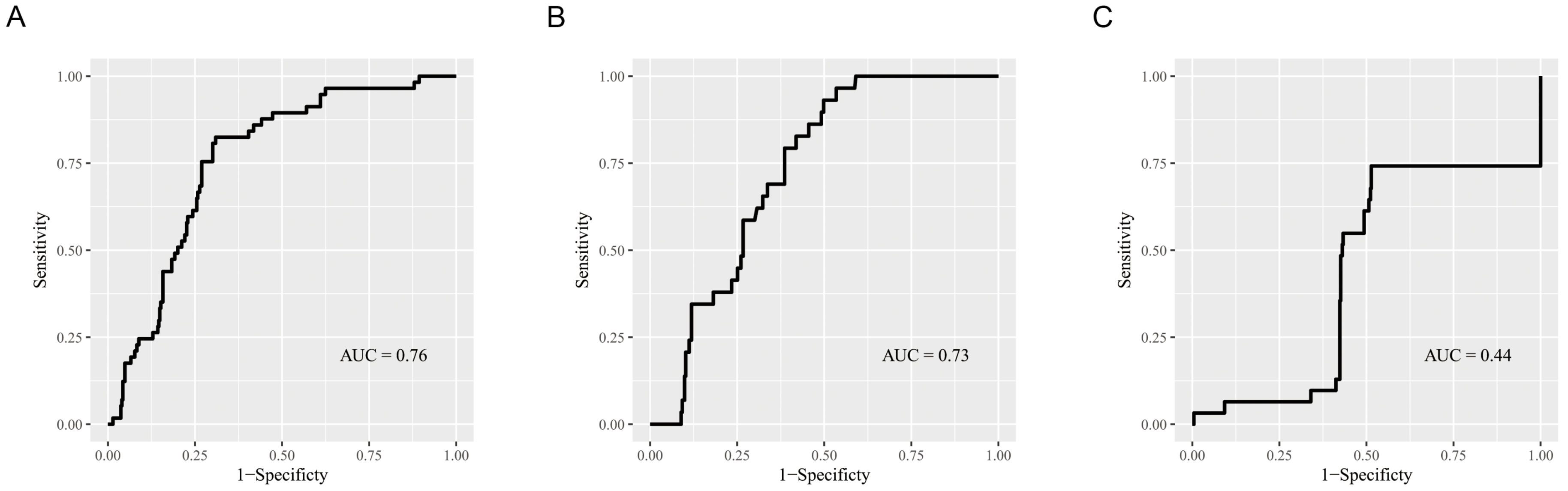

Supplementary Figure 7. AUC curves of the model based on clinical metadata on prediction of glaucoma progression. a to c: predictive performance of the model in the validation set, and external test set 1, 2.

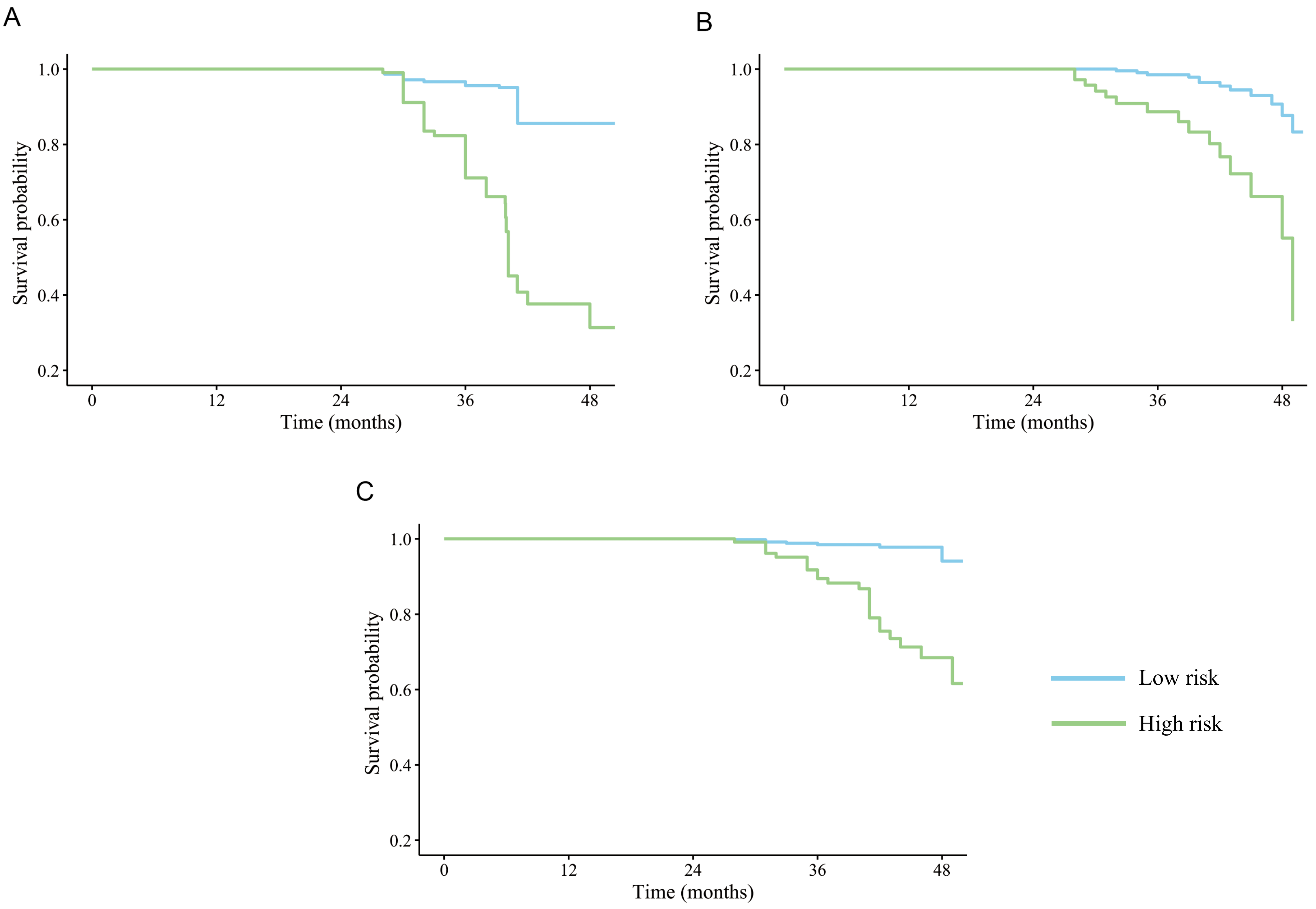

Supplementary Figure 8. Kaplan-Meier curves for predicting glaucoma progression accuracy

a to c: predictive accuracy in the validation set, and external test set 1, 2. Survival curves in blue and green represent the high-risk and low-risk subgroups stratified by the upper quartile. P value is computed using a one-sided log-rank test between the two subgroups, and all P values are less than 0.001.

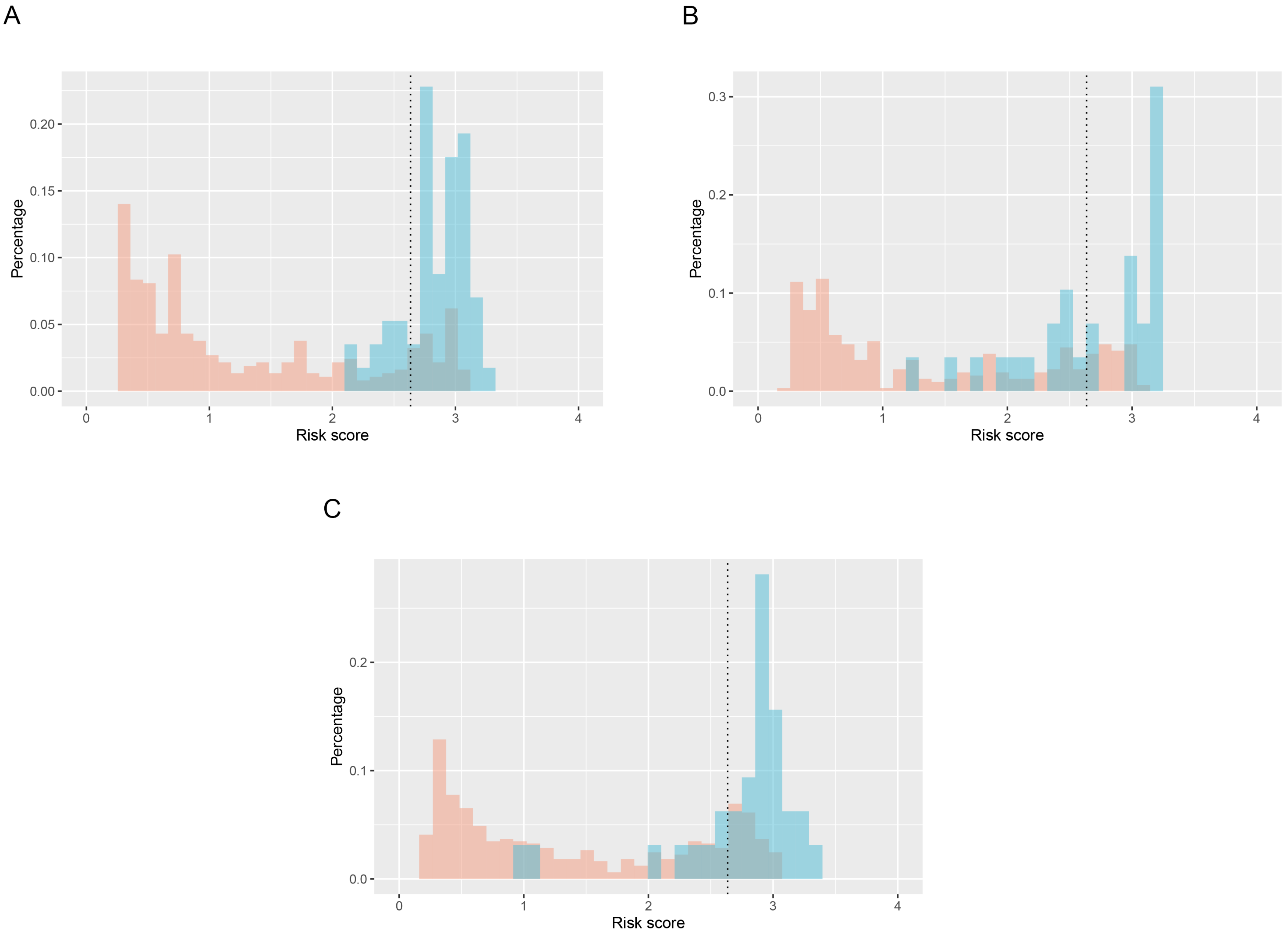

Supplementary Figure 9. The distribution of risk scores of the predictive models across all datasets in the prediction of glaucoma progression

The black dot line represents the low-high threshold of risk score (2.6352). The red bars represent the proportion of eyes without glaucoma progression, while the blue bars represent the proportion of eyes with glaucoma progression. a to c: glaucoma onset in the validation set, and external test set 1 and 2.

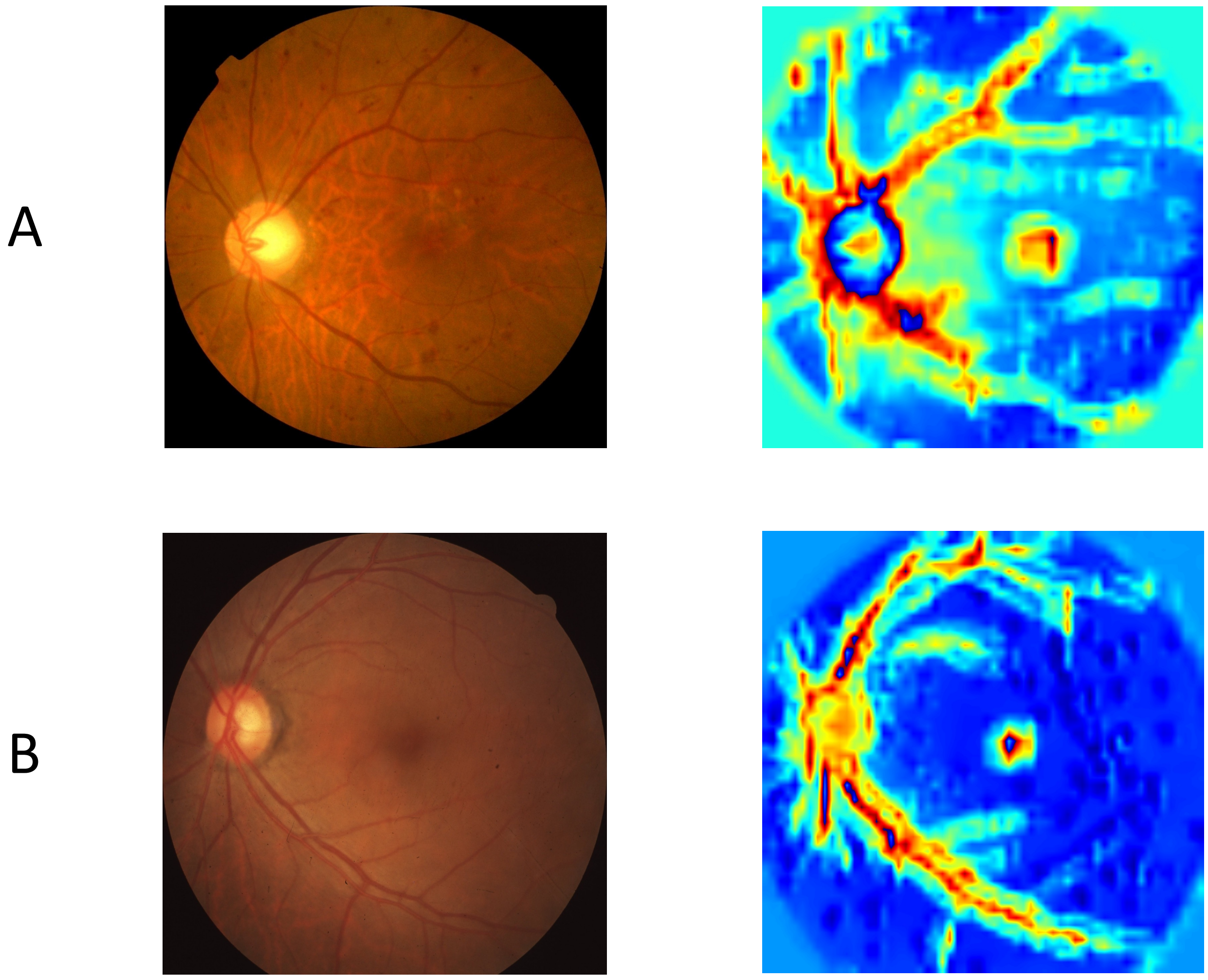

Supplementary Figure 10. Saliency maps of the deep learning models to diagnose glaucoma

Visual explanation of the key regions the models used on diagnostic predictions. a and b: the heatmaps of the typical samples of eyes with (a) and without (b) possible glaucoma. The saliency maps suggest that the AI model focused on the optic disc rim and areas along the superior and inferior vascular arcades, which are consistent with the clinical approach whereby nerve fiber loss at the superior or inferior disc rim provide key diagnostic clues.

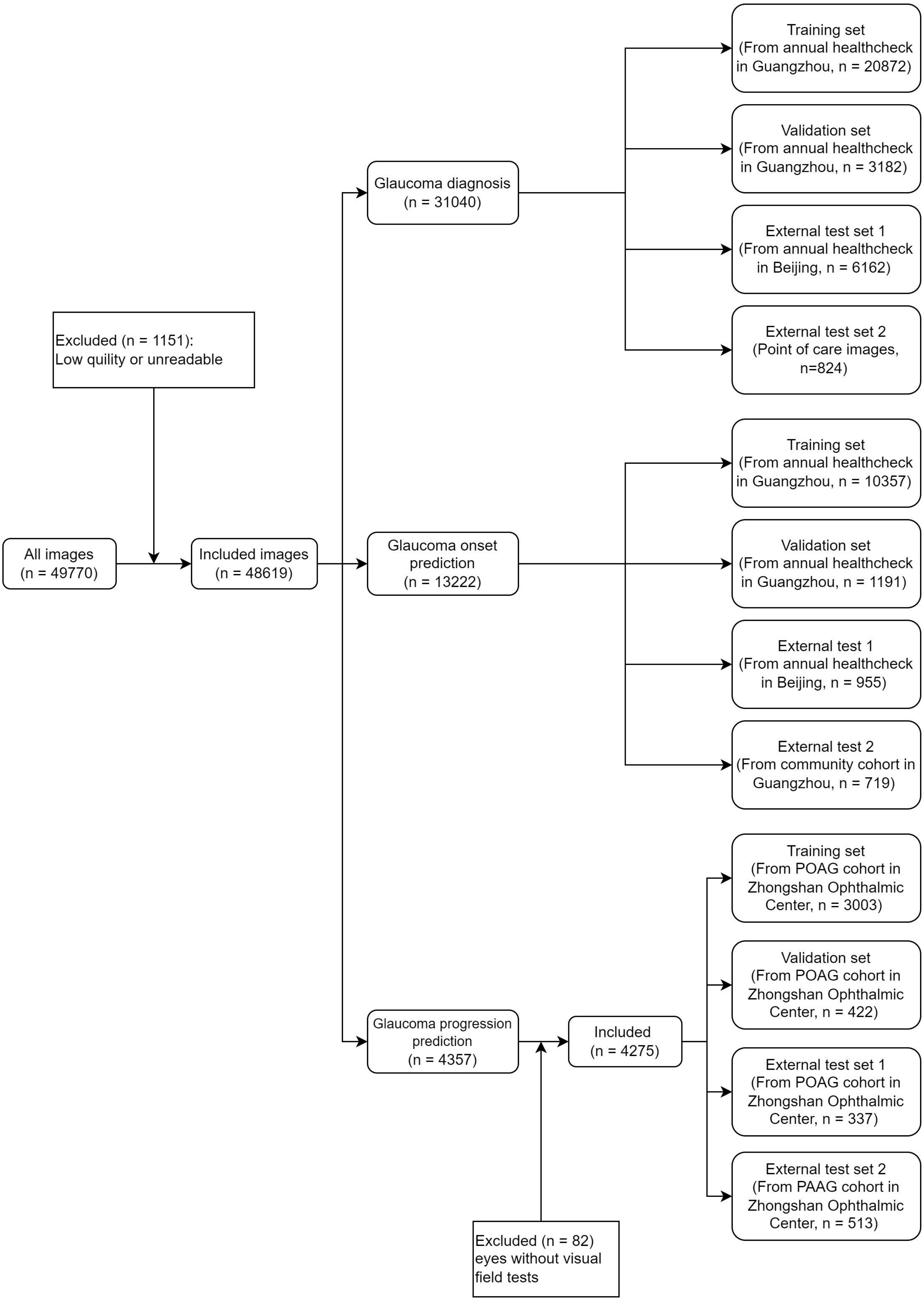

Supplementary Figure 11. Flow chart of image quality control and the datasets used in the current study

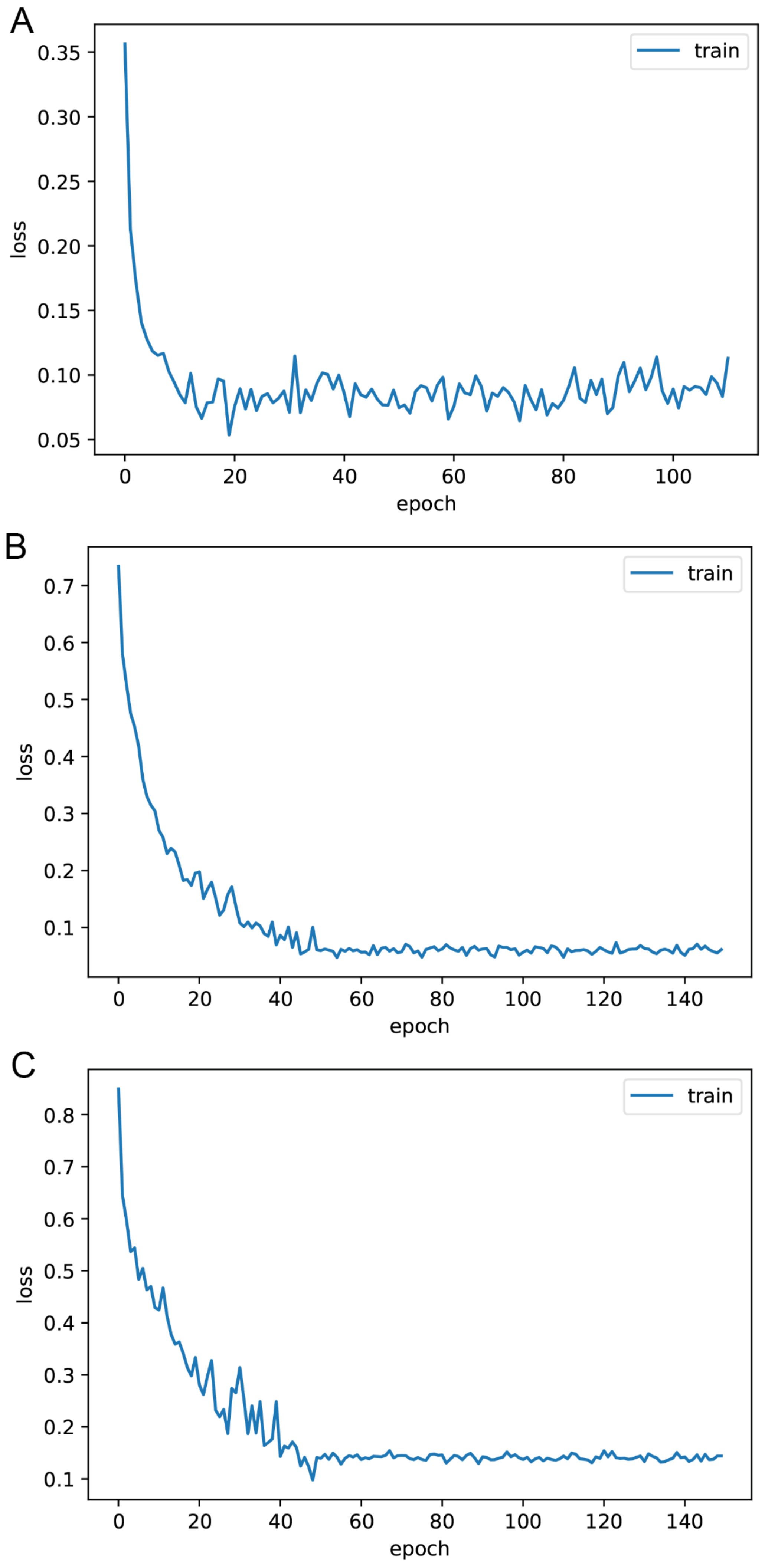

Supplementary Figure 12. Loss curves during model training in Task 1 and 2
a: loss curve of glaucoma diagnosis; b: loss curve of glaucoma onset prediction; c: loss curve of glaucoma progression prediction.

Supplementary Table 1. Performance of automatic retinal structure segmentation

**Structure**	**No. of training images**	**No. of validation images**	**IOU score in the validation set**
Optic disc	1853	486	0.847
Optic cup	1860	463	0.669
Macula	1695	423	0.570
Blood vessels	160	40	0.538

Supplementary Table 2. Incidence rates of glaucoma in the validation and the external test sets according to two-strata of the deep learning model

Risk group	Number of eyes	Number of events	Incidence rate
Validation set			
Low risk	892	2	0.2%
High risk	299	15	5.0%
External test set 1			
Low risk	686	4	0.6%
High risk	268	15	5.6%
External test set 2			
Low risk	524	2	0.4%
High risk	194	8	4.1%

Supplementary Table 3. Comparison of AUCs of the predictive algorithms in the subgroups stratified by age, sex and disease severity

Incidence prediction		Validation set			External test set 1			External test set 2	
	Sample size	AUC (95%CI)	P value	Sample size	AUC (95%CI)	P value	Sample size	AUC (95%CI)	P value
**Age**									
<60	602	0.96 (0.91-1.00)	0.15	563	0.95 (0.87-1.00)	0.22	405	0.82 (0.63-1.00)	0.30
≥60	590	0.83 (0.66-1.00)		392	0.87 (0.77-0.97)		314	0.92 (0.84-1.00)	
**Sex**									
Male	526	0.87 (0.72-1.00)	0.59	482	0.94 (0.87-1.00)	0.28	380	0.88 (0.80-0.96)	0.19
Female	592	0.93 (0.82-1.00)		473	0.87 (0.77-0.98)		339	0.96 (0.88-1.00)	
**Progression prediction**		**Validation set**			**External test set 1**			**External test set 2**	
	**Sample size**	**AUC (95%CI)**	**P value**	**Sample size**	**AUC (95%CI)**	**P value**	**Sample size**	**AUC (95%CI)**	**P value**
**Age**									
<60	366	0.89 (0.84-0.93)	0.87	304	0.89 (0.84-0.94)	-[#]	424	0.90 (0.85-095)	0.28
≥60	56	0.88 (0.84-0.94)		33	-[#]		89	0.79 (0.59-0.99)	
**Sex**									
Male	152	0.91 (0.88-0.95)	0.08	152	0.86 (0.77-0.94)	0.51	293	0.84 (0.76-0.93)	0.08
Female	270	0.97 (0.92-1.00)		185	0.82 (0.75-0.89)		220	0.93 (0.89-0.97)	
**Disease Severity**									
MD≥-6 dB	380	0.93 (0.90-0.95)	0.004	297	0.86 (0.80-0.92)	<0.001	433	0.88 (0.80-0.95)	0.37
MD<-6 dB	42	0.69 (0.55-0.84)		40	0.99 (0.99-1.00)		80	0.80 (0.66-0.94)	

*Comparison of AUCs between groups using Delong's test. AUC, area under curve. MD, mean deviation; CI, confidence interval.

[#]The AUC is not calculable here since there is no positive case.

Supplementary Table 4. Progression rates of glaucoma on the validation and the external test sets according to two-strata of the deep learning model

Risk group	Number of eyes	Number of events	Incidence rate
Validation set			
Low risk	316	12	3.8%
High risk	106	45	42.4%
External test set 1			
Low risk	266	12	4.5%
High risk	71	17	23.9%
External test set 2			
Low risk	397	8	2.0%
High risk	116	23	19.8%

Supplementary Table 5. Details of the DiagnoseNet and PredictNet including key hyperparameters and average running time for training, validation, and testing phases

	Task	Diagnosis	Incidence prediction	Progression prediction
**Model Details**	No. of parameters (M)	4,057,253	652,578	652,578
	Model size (MB)	48	7.6	7.6
**Training Phase***	Batch size	64	64	64
	Running time per epoch (s)	1110	18	9
	No. of epochs	11	37	11
	Overall time (minutes)	203.5	11.4	1.8
**Validation Phase***	Batch size	64	64	64
	Running time per test sample (ms)	46.88	3.328	3.328
**Testing Phase***	Batch size	64	64	64
	Running time per test sample (ms)	46.88	3.328	3.328

*The models were trained and validated with GPU: NVIDIA Tesla v100 16GB x 8, CPU: x86_64 72 Core, and RAM: 644GB.
